# Retraction: SALL4 as an Epithelial-Mesenchymal Transition and Drug Resistance Inducer through the Regulation of c-Myc in Endometrial Cancer

**DOI:** 10.1371/journal.pone.0298990

**Published:** 2024-02-13

**Authors:** The PLOS ONE Editors

Following publication of this article [1] concerns were raised regarding Figs 1, 2, and 5. Specifically,

- In Fig 1A, there appears to be a vertical discontinuity between the N5 and T5 lanes of the GAPDH panel.
- In Fig 2B the lower right region of the Ishikawa SALL4A panel appears to overlap with the top left region of the Ishikawa SALL4B panel.
- In Fig 2E, the following Migration panels appear to overlap despite representing different experimental conditions:
  ⚬ The top region of the Ishikawa 48 h vector panel and the bottom region of the AN3CA 48h SALL4-sh1 panel
  ⚬ The bottom region of the Ishikawa 48h SALL4A panel and the top region of the Ishikawa 48h SALL4B panel
  ⚬ The bottom region of the Ishikawa 48h SALL4B panel and the top region of the AN3CA 48h Scr-sh panel.
- In Fig 2E, the bottom left region of the AN3CA SALL4-sh1 Invasion panel appears similar to the AN3CA SALL4-sh2 Invasion panel.
- In Fig 5F lane 1 of the SALL4B panel appears similar to lane 2 of the E-Cadherin panel when vertically flipped.

The corresponding author stated that the majority of underlying data for this article are no longer available, but they provided blot images in support of results shown in Fig 1 and Fig 2, replacement panels for Fig 2B and 2E, and replication data in support of Fig 5F. Regarding the concerns in Figure 2B and Fig 2E, the corresponding author stated that errors were made during figure preparation.

Whilst the blot data provided for Fig 1A did resolve the concerns regarding the apparent vertical discontinuity, the images provided for the remaining western blot data in Figs 1 and 2 raised additional questions regarding the underlying data pertaining to this article.

Additionally, while the replacement images for Fig 2 provided post-publication lend support for the reported findings, the *PLOS ONE* Editors remain concerned about the extent of figure concerns in this article, the unavailability of the original underlying data, and the implications of these issues for the overall reliability of data reporting. Therefore, the *PLOS ONE* Editors retract this article.

XX did not agree with the retraction. LL, JZ, XY, CF and HX either did not respond directly or could not be reached.
